# Women Tend to Defect in a Social Dilemma Game in Southwest China

**DOI:** 10.1371/journal.pone.0166101

**Published:** 2016-11-09

**Authors:** Riccardo Pansini, Lei Shi, Rui-Wu Wang

**Affiliations:** 1 State Key Laboratory of Genetic Resources and Evolution, Kunming Institute of Zoology, Chinese Academy of Science, Kunming, 650223, China; 2 Statistics and Mathematics College, Yunnan University of Finance and Economics, Kunming, 650221, China; 3 Center for Ecological and Environmental Sciences, Northwestern Polytechnical University, Xi’an, 710072, China; Tianjin University of Technology, CHINA

## Abstract

Cooperation theories assume that interacting individuals can change their strategies under different expected payoffs, depending on their social status or social situations. When looking at sex differences in cooperation, the existing studies have found that the genders cooperate at similar frequencies. However, the majority of the data originate within Western human societies. In this paper, we explore whether there are gender differences in cooperation in China. An Iterated Prisoner’s Dilemma game with a punishment option was used to gather data about Southwest Chinese subjects in a culture in which men have a hierarchical advantage over women. Results indicate that men invested into partners significantly more than women did (34% ♂ vs. 24% ♀) while women, in turn, were more likely to defect (65% ♀ vs. 50% ♂). In this region, women have customarily held less economic power and they are used to obtain a payoff typically lower than men. We suggest that the women’s willingness to invest in cooperation has decreased throughout evolutionary time, providing us with an illustration of a culturally-driven shift towards a disparity in gender cooperation interests.

## Introduction

Within the behavioural sciences, game theory and the Prisoner’s Dilemma game have provided key insights aiding in describing the conundrum faced by unrelated individuals who can either profit from cooperation [1] or may be able to further profit by defecting which, in effect, means turning against their partners [2, 3]. To increase their personal fitness, self-interested players are expected to prefer higher payoffs before engaging in costly interactions (e.g. [4]). Yet, departing from this framework, there is extensive evidence that cooperation in humans doesn’t necessarily follow this general rule (e.g. [5]).

In anonymous, one-shot and Iterated Prisoner’s Dilemma games [6, 7], when a dyad of cooperators act together, their individual fitness increases. When a cooperator and a defector are paired, the cooperator is exploited and its fitness decreases, while concurrently the defector’s fitness increases more than in the former pairwise cooperation setting. When two defectors act together, instead, no novel contribution is produced and there is no payoff. In one-off interactions, defection proves to be a better strategy for rational agent models (i.e., homo economicus) to choose, but in the long run—that is, in repeated interactions—it becomes harder to justify. This paradox is solved by adopting reciprocal cooperation that leads to repeated mutual investments [8]. However, if the likeliness of obtaining a reward from the cooperative interaction varies amongst the players depending on their individual characteristics, investments are also hypothesised to change [9, 10] and, specifically, to decrease for those players whose expected payoffs are low.

The individuals’ choice between the temptation to defect or the investment toward cooperation can specifically depend on: (a) the social status of the subjects and (b) the social context in which the subjects live [11]. Yet, to date, it is still not clear to what extent these two elements may practically condition the outcome of cooperation. Here, we examine human cooperation whilst specifically accounting for these differential characteristics of the subjects, based on the assumption that the nature of the social relationships in which individuals typically engage is, in effect, constantly ruled by an asymmetry of resources and the exercise of power [12–16].

We may well expect an inherent power asymmetry when the two genders interact [17], independent of culture and geography [18, 19]. Both physical and psychological differences lead to an unbalanced weight between the genders that can easily modify the outcome of the cooperation game. To discern whether in social dilemma games there is a gender differentiation in cooperation across humans, Croson and Gneezy [20] had initially concluded that the results of 18 games they analysed were contradictory. Later, though, Balliet and colleagues [21] collated 50 years of game theory experiments, providing the largest meta-analysis to date, incorporating 272 Prisoner’s Dilemma, Public Good, and Resource Dilemma games into their study. These authors concluded that, from a geographical point of view, the initial asymmetry found between the genders cancels out across cultures, resulting in similar levels of cooperation between men and women worldwide. In Balliet et al. [21], though, data from the Far East were few, suggesting that this meta-analysis may have missed important trends stemming from cultures uniting a great amount of people and yet clearly distinct from the rest of the world. Limited to Far East Asia, eleven studies from Japan, one from Singapore and one from Taiwan found no evidence in these politically and economically different regions of any difference in cooperation between men and women. The same conclusion of Balliet and colleagues was also reached by Falk and colleagues in a recent global survey [22]. For the first time, here, we obtain discordant results originating in the historically and culturally most influential country in this Far East region, China.

The Chinese society provides a classical example of a heterogeneous social system where gender roles are still distinctly separated, especially in the developing areas of this country. Gender separation is still an important social norm in some areas of China, regardless of the original guidelines put forth by the Communist Party [23]. At present, this gender difference remains visible in daily life (see further reasoning in S1 File). We were, therefore, not sure whether men and women set the same cooperative trend frequently occurring in other countries.

We conducted experiments with students who were asked to cooperate, defect, or punish, in an iterated Prisoner’s Dilemma game similar in design to those used by Dreber et al. [24] and Wu et al. [25]. In this study we focus our attention mainly on cooperation and defection, the latter behaviour of which the female students chose at an unexpectedly high rate.

## Methods

Between 2010 and 2013, students with no prior experience of game theory and behavioural economics tests agreed to be subjects for experimentation. Informed consent forms were obtained by all participants and the experiment was approved by the Yunnan University of Finance and Economics Ethics Committee on the use of human subjects in research. The students were all undergraduates, and were enrolled in different majors at either: (1) Yunnan University, (2) Yunnan University of Finance and Economics, or (3) Yunnan Agricultural University, all institutions based in the city of Kunming. The home cities of the students were widely dispersed across the southwest province of Yunnan and further across China (60% students were born in Western China). The three universities are respectively ranked within different leagues in China in terms of education and research standards. Their tuition fees, also, varied from relatively more to less expensive. The sample group of students was therefore comparatively representative of the different range of social classes found in China engaged in both humanities and science subjects.

The students were individually assigned to a computer in a visually partitioned computer lab. After receiving brief instructions as to how to play the game, they were left to anonymously interact with partners sitting on other computers in the same room. Each computer was connected via Local Area Network to a master machine which provided the players with timely, repeated Prisoner’s Dilemma rounds through z-Tree [26]. The subjects were free to choose from three strategies: cooperation, defection, or punishment. The different payoffs to each player in every two-player game are reported in the table below (Table 1).

**Table 1:** 

	Cooperation	Defection	Punishment
Cooperation	**1, 1**	**-2, 3**	**-5, 1**
Defection	**3, -2**	**0, 0**	**-3, -2**
Punishment	**1, -5**	**-2, -3**	**-5, -5**

A total of 240 students, 125 men and 115 women, aged between 17 and 24, participated this study. Nine replicated sessions involved different numbers of men and women with an average of 26.6 subjects each (S.E. = 1.1). Each participant took part only once. The individuals were assigned to dyads which repeatedly interacted with each other during iterated PD “rounds”; the dyads changed instead randomly at different “interactions”. After each round was complete, we set the probability of a new round to take place with the same partners at 0.75 (following the same "shadow of the future of Dal Bò [27]). To allow for uncertainty about the repeated round with the same partners, each subject was not told how many rounds were involved in each pair’s interaction. During each round of a game, the two participants simultaneously chose one of the three available options. After the completion of each round, the choices made were shown to the participants together with the updated payoff scores.

We analysed 4928 behavioural responses, specifically a total of 1750 rounds grouped in an average of 20.56 interactions at each replicate session. The duration of each replicate session was approximately 1.5 hours. The average payment each subject received was 28.3 Yuan Renminbi (RMB) determined by the total of their accumulated points multiplied by RMB 0.2 (see further details in S1 File).

Since the data present non-constant variability and we can expect a priori the subject behaviour to be partly correlated and heterogeneous, the results were analysed by Generalized Linear Mixed Models in R 3.2.4 [28] with the lme4 package [29].

Written informed consent forms were obtained by all participants. The experiment was approved by the Yunnan University of Finance and Economics Ethics Committee on the use of human subjects in research and carried out in accordance with the approved guidelines.

## Results

In respect to cooperation, defection, and punishment, irrespective of gender, Americans [24] exhibited each behaviour with a frequency of 52.4%, 40%, and 7.6%, respectively. In our experiment, we found instead 29.03%, 57.4%, and 13.57% frequency rates for the same three behaviours. Chinese students therefore chose to cooperate on average 23.37% less than the Americans. Chinese students chose to defect on average 17.4% more frequently than the Americans, and to punish 5.97% more frequently.

**Fig 1:**
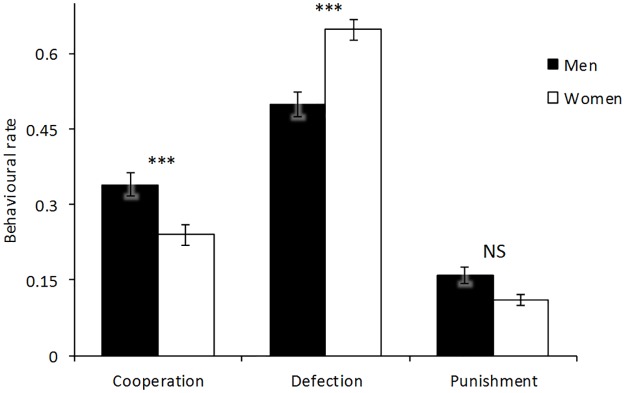
Proportions of behavioural responses between participants. Men were more cooperative (34% vs. 24%) and women more likely to defect (65% vs. 50%) (*** is p ≤ 0.001). Instead, there was no difference between the genders (16% vs. 11%, NS) in respect to punishment (w = 7878.5, *p* = 0.1984). Error bars represent Standard Errors of the means.

Before the experiments, we obtained several individual variables from the students. Gender significantly explained variation in defection rates. As visualised in Fig 1, women consistently defected more than men (F_0,1_ = 10.1331, S.E. = 1.859, *p* = 0.00154), (see Table A in S1 File for the full output of the GLMM).

**Fig 2:**
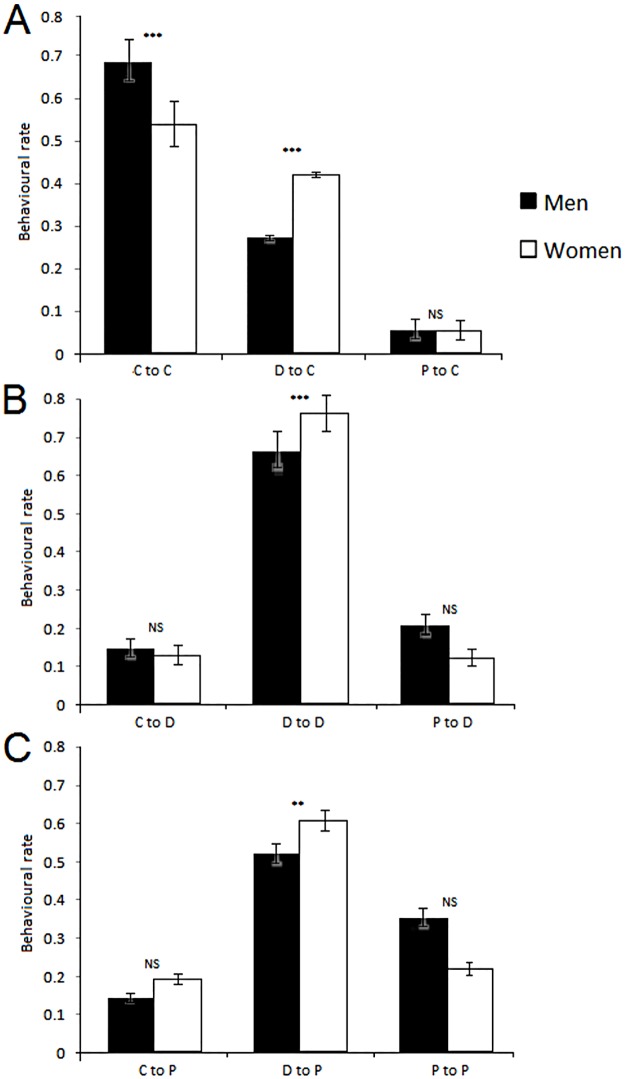
Histogram plots of the first order conditional strategies used at the following round in response to Cooperation (A), Defection (B), and Punishment (C). Error bars represent Standard Errors of the means. The statistical output is reported in Table B in S1 File.

To further test for differences between the sexes, we ranked all participants with respect to their consistency in replicating cooperation, defection, or punishment responses (Fig 2). Participants cooperated and defected at different rates according to their gender. There was no significant difference between the genders for choosing punishment nor in the ultimate financial gain obtained at the end of the game (Mann-Whitney non-parametric tests: w = 6647.5, *p* = 0.3153). Nevertheless, men collected higher earnings when defecting (F_0,1_ = 113.4, S.E. = 0.113, *p* = 0.0042). Both genders obtained higher earnings when cooperating (F_0,1_ = 327.2, S.E. = 0.006, *p* = 0.0001) but lower earnings when punishing (F_0,1_ = 3901, S.E. = 0.056, *p* = 0.0412).

**Fig 3:**
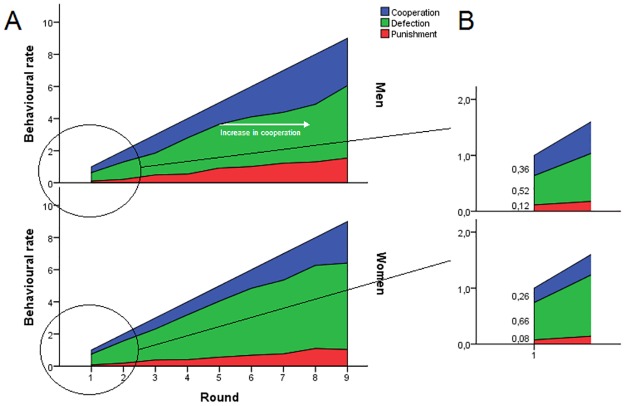
**A** Iterations of the Prisoner’s Dilemma game. Each interaction had a maximum of nine repeated rounds with the same dyad of players. In such cases, nine behavioural strategies were required to be chosen in succession. The cumulative rate of cooperation, defection, and punishment are reported in different colours in stacked up plots. Defection, in green, was used by women extensively, from the first round onwards at a constant rate. Men instead cooperated at relatively higher rates with the same partners from the fifth round on. **B** Magnification of the first round. Without prior knowledge of the other player’s behaviour, women defected more frequently than men.

During each game with the same partners, on average men began to choose to cooperate at relatively higher frequencies from the fifth round. This trend was not apparent in women, who tended to defect throughout (Fig 3A). Moreover, during the first round, without a previous round of experience of their partner’s behaviour, women defected 14% more often than men (Fig 3B).

Looking at first order conditional strategies and their corresponding disparities between genders, we answered the question of how men respond to cooperation, defection, or punishment differently from women (Fig 3). When participants received a cooperation response choice from their partners, during the following round with the same partner male subjects cooperated more often than female ones (*p* = 0.0022, Mann-Whitney test) and female subjects defected more frequently than male ones (*p* = 0.0022). No differences, however, were found in response to punishment (*p* = 0.37). In relation to defection received from the previous round, although no gender differences were found directly in the rates of cooperation and punishment (*p* = 0.71 and *p* = 0.073), female participants nonetheless used this strategy more commonly than men (*p* < 0.001). Finally, in relation to punishment received, although no gender differences were identified in concurrence with cooperation and punishment (*p* = 0.16 and *p* = 0.052), female participants still used this strategy more than men did (*p* < 0.011). The statistical output is reported in Table B in S1 File.

We accounted for variation in the behaviour of research subjects to attempt to compare conformity in the West and in China. The Standard Deviation of the overall monetary payoff of American students used for a similar experiment (S. I. of [24]) had Standard Deviation values ranging from 15 to 25. The inter-individual variation across our subjects was lower (S.D. = 5.469 to 7.156), suggesting that Chinese students of both genders behaved similarly to one another, showing little individual variation.

Finally, we fitted a model to the overall monetary payoffs to check if the use of punishment could explain higher earnings. Similarly to Dreber et al. [24] and Wu et al. [25], we also found that ‘winners’ were less likely to punish. Both men and women decreased their monetary gains when adopting punishing strategies (quantile regression model to the average gains, combined with the three strategies of cooperation, defection, and punishment; *t* = -10.818, *p* < 0.0001).

## Discussion

In sharp contrast to Western and other Asian women [21, 30], our Chinese female subjects (students who originate mainly from Southwest China) round after round consistently failed to select cooperation, instead preferring defection in an anonymous Prisoner’s Dilemma game. Male subjects, however, were significantly more likely to cooperate. This holds true throughout the on-going rounds of the strategy game, in reaction to responses from previous rounds. This behaviour is in line with the findings of Gaechter and Poen (pers. comm.), who surveyed men and women in Public Goods games and concluded that women do not substantially calibrate their responses to the behaviours received as they play the game over time.

As in any human society, women in Southwest China are plainly dissimilar to men, largely due to their physical differences and their role as mothers. In exchange for being more attractive partners and better mothers, women can, in fact, show an open, indulgent and empathic attitude [31, 32]. Evidence indicates now that the inner characteristics of the social network of women augment cooperation. Women have, in fact, been found to have fewer personal connections but ones of higher value than men [33, 34]. Further, in hunter-gatherer societies women induced families to cooperate outside their inner circle for social benefits [35].

In most situations, though, gender difference does not lead to different levels of cooperation. In traditional Chinese society, conversely, different cooperation output is quite likely due to the instilled gender roles. We analyse three factors underpinning such a gender gap: (a) women’s literacy, (b) their employment rates and relatively lower salaries, and (c) their negligible representation at higher political levels. (a) The majority of young Chinese women with access to secondary and higher education is only 36.2% [36]. (b) Chinese women are paid on average 75% of men for the same job position due to gender discrimination, as compared to 84% of men’s wages for the same work globally [37]. (c) At the highest political levels in China, of the leading roles controlled by the government, only 2.72% are held by women [38].

It is apparent that Chinese women do not partake in the same hierarchical class as Chinese men. As a consequence, the female students monitored in this study seemed to not expecting to achieve the same level of cooperative return as their male counterparts, especially in an economic context such as the one which we modelled. As shown by the results presented here, by mainly defecting, Southwest Chinese students incur lower and ‘safer’ overall costs when interacting with other anonymous partners. This conclusion complies with the principle that, at times, men decrease their risk aversion due to greed, whereas women increase aversion. This appears to be a fear-driven response of losing their cooperation investment due to the exploitation by others of their contribution [39, 40].

We hypothesise that if gender asymmetry had not developed in such a skewed fashion over social evolutionary time, our subjects would perhaps cooperate more. Two recent researches focussing on the Mosuo minority in the same province our study took place in, Yunnan, support our hypothesis. The Mosuo minority matriarchal society leaves it up to women to mainly manage family resources (e.g. [41]) and therefore they hold relatively more power than most other Chinese women. Mosuo women were found significantly less risk averse than neighbouring communities, when having to invest a certain endowment into a Lottery game [42]. In a Dictator game context, when women are normally more willing to donate more than men, in the Mosuo the trend is reversed [43]. The Mosuo are thus a Chinese community with economic behaviour less skewed between the sexes and tending more to the Western standard. This trend was previously confirmed in another matriarchal society, the Indian Khasi [44].

Our results should not be interpreted as a demonstration of feminine, self-centred behaviour. In fact, across China, when asked to measure themselves in association with their family and friends, women think relatively more in holistic terms than men (S. I. of [45]). Combining our findings with those of Talhelm and colleagues, our data indicate that although it does not often pay for Chinese women to cooperate with other anonymous partners, they may still retain a strong feeling of community association. A large literature shows that with more diverse ties formed within a heterogeneous social network [46–55], higher social and economic development will result. Knowing the authorities appreciate significance of gender imbalance issues in China, and that awareness of the sex ratio level in the current population of reproducing age [56], it is expected that investment in gender equality programmes could produce considerable benefit for this type of traditional society.

## Supporting Information

S1 File Supporting Information. (DOCX)

S2 File Supporting Data. (XLSX)
