# Supplementary material for: Women Tend to Defect in a Social Dilemma Game in Southwest China
Source: PLoS One. 2016 Nov 9;11(11):e0166101. doi: 10.1371/journal.pone.0166101 (PMC5102488; doi:10.1371/journal.pone.0166101)
Supplement: S1 File — (DOCX) [file pone.0166101.s001.docx]

# Women tend to defect in a social dilemma game in Southwest China

Riccardo Pansini, Lei Shi, Rui-Wu Wang

r.pansini@gmail.com

# Supporting information

## Supplement to Introduction

Game theory experiments have mostly been performed in western countries, where subjects are exposed to cultures promoting the individual ([Henrich et al. 2010](#_ENREF_15)). In Asia, people predominantly think holistically about themselves, as integrant part of the social communities they belong to ([Nisbett et al. 2001](#_ENREF_25)). By all odds, the aspect researchers try to unravel in these cooperation studies is exactly how individuals balance their own, selfish benefit at the stake of the benefit of the collectivity ([Markus & Kitayama 1991](#_ENREF_22)). It is therefore significant to test for differences in cooperation strategies displayed by members of western countries and members of societies with a stronger collectivist tradition ([Cox et al. 1991](#_ENREF_8)).

Even more so than other Eastern Asian countries ([Oyserman et al. 2002](#_ENREF_26)), China is a country with a long tradition of portraying the accomplishments of the society as such ([Yang 1995](#_ENREF_32)). For centuries, this country was ruled by an oligarchy of slowly shifting dynasties. Also in the course of the last century, as a republic, the nation has conserved a noticeable class segregation, once commanded by the Confucian doctrine. The social classes were for long divided broadly into a limited elite and a large proletariat. This situation has changed lately, due to the fast rising Chinese economy. The last generation has appreciated the rise of a middle class ([Bian 2002](#_ENREF_2)), which increased at a fast pace till present time. The extent of this middle class is now over one third of the size of the worldwide middle class, characterised by an income between USD 10,000 and 100,000 ([Davies et al. 2013](#_ENREF_10)). Some suggest that this collectivist culture could be at stake and perhaps slowly turn into a more individualistic one with the single person being the gravitational centre ([Nee 1996](#_ENREF_24); [Yang 1996](#_ENREF_33)).

We performed our research in the province of Yunnan, a south-west region situated at the border with Burma, Laos, and Vietnam. This province is home to many Chinese minorities beside the still predominant Han ethnicity. The people here are still considerably more traditional than in the eastern, economically more advanced areas of China ([for an economic distinction of the areas, see Walder 2002](#_ENREF_28)). We therefore predicted the participants to our experiments to behave according to what portrayed by the traditional Chinese conventions. Regardless of their ethnic origin, we expected the majority of our participants to originate from families with a predominantly patriarchal tradition and strong families ties ([Yifei 2011](#_ENREF_34)).

With the advent of the communist party in 1949, it gradually looked like women were given empowering opportunities to hold responsibility jobs. In reality, often justifying physical differences, men have always been on a higher step in the struggle for independence and self-maintenance ([Nee 1996](#_ENREF_24)).

A new study by Cárdenas et al. ([2014](#_ENREF_4)) found that Colombian girls cooperate significantly less the boys but using a different experimental setup resembling a PD game. While at school, the children aged 9-12 were asked to perform a physical education exercise in which they were free to place balls in private bins or shared bins between two children and obtain stationery at the end. In that case, the girls acted more “selfishly” than boys did. No gender difference was found when the study was replicated with Swedish children, instead.

## Supplement to Results

To further test for differences between the sexes, we ranked all participants in respect to their consistently replicating cooperation, defection, or punishment responses. Participants cooperated and defected at different rates according to their gender (Figure S1).


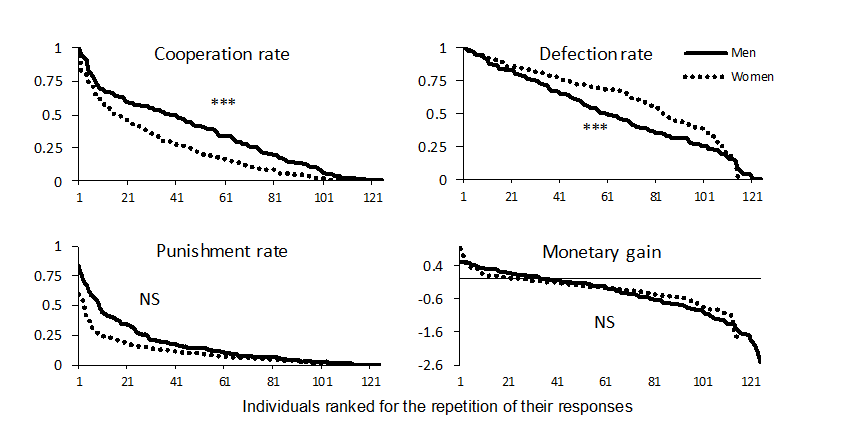


**Figure A** Behavioural frequencies of all 240 participants, 125 men and 115 women. The individuals that consistently repeated their responses are thus positioned at the far left and far right sides of the *x* axes; whilst at the centre are those individuals that gave the most varied responses. Men were more cooperative and defected less than women (*** is p ≤ 0.001); there was no difference between the sexes for punishment (NS). The fourth graph shows the overall monetary payoff rate at the end of each game which showed no significant difference (NS).

By ordering the sessions with respect to the genders’ different ratios, we could show the effect of gender in our results. At higher number of females in respect to males, the overall cooperation of the groups significantly decreased (correlation coefficient 0.96, *p* < 0.0001, Spearman rank correlation *p* = 0.0011; see Figure S1). This is a supplementary indication showing the effect of the defective strategy of women on the whole groups of interacting students.

Figure B Decrease in cooperation frequency at higher women numbers relative to men. The more female students participated in the experiments, the lower cooperation was attained at each experiment. The intercept line is y = -0.1457x + 0.4567, with R² = 0.606.

We found an effect of age in the behaviour of our subject. In particular, women defected more at older ages (Figure S2).


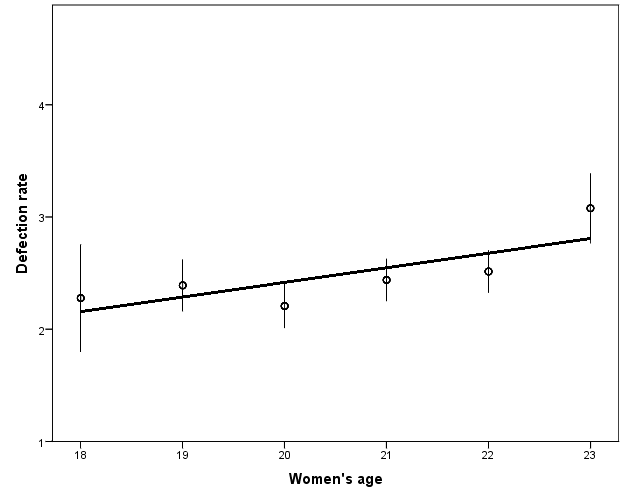


Figure C The defection rate of women is higher at older ages (the GLMM analysis reported in Table S1). The age of female participants has been plotted against increasing defection rates, expressed in points that the students lost by choosing defection.

The table below shows the full output of the GLMM analysis conducted on the participants.

| **Defection rates** | Estimate | df | Sum Sq. | SE | F | *p* |
| --- | --- | --- | --- | --- | --- | --- |
| Gender | 3.12771 | 1 | 50.551 | 1.85971 | 10.1331 | 0.00154 |
| Age | 0.13537 | 1 | 4.378 | 0.06635 | 0.8776 | NS |
| Gender * Age | -0.16959 | 1 | 18.031 | 0.08921 | 3.6143 | 0.015 |

Table A Results of the GLMM on the defection rates of the participants to the experiments. The random factors included the experiment’s participants. The significant Age covariate and the 2-way factor of Gender combined with Age show that females defect more than male participants did, especially at older ages.

| Response strategy | Response strategy  Average numbers | | Standard Errors | | Mann-Whitney test |  |
| --- | --- | --- | --- | --- | --- | --- |
|  | Men | Women | Men | Women | *p-value*s | |
| $C\text{ }to\text{ }C$ | 14.66 | 9.88 | 1.10 | 1.23 | 0.002209 | |
| $D\text{ }to\text{ }C$ | 5.78 | 7.71 | 0.69 | 0.59 | 0.002205 | |
| $P\text{ }to\text{ }C$ | 1.10 | 0.92 | 0.12 | 0.18 | 0.3751 | |
| $C\text{ }to\text{ }D$ | 4.58 | 4.61 | 0.44 | 0.50 | 0.7198 |  |
| $D\text{ }to\text{ }D$ | 21.49 | 28.21 | 1.15 | 1.01 | 3.78 x 10^-5^ |  |
| $P\text{ }to\text{ }D$ | 6.56 | 4.40 | 0.66 | 0.45 | 0.07381 |  |
| $C\text{ }to\text{ }P$ | 1.45 | 2.12 | 0.24 | 0.37 | 0.1681 |  |
| $D\text{ }to\text{ }P$ | 5.59 | 6.95 | 0.51 | 0.57 | 0.011 |  |
| $P\text{ }to\text{ }P$ | 3.73 | 2.47 | 0.43 | 0.28 | 0.05228 |  |

**Table B** Results of the first order conditional strategies used at the following round and their corresponding disparities between genders.

## Supplement to Discussion

The empirical models we adopted to study cooperative behaviour in Chinese people returned results that were strikingly different from what previous studies detected across the globe ([Balliet et al. 2011](#_ENREF_1)). In relation to punishment, Chinese people have previously been found to exhibit low levels of punishment, which does not concur with that demonstrated in other authoritarian countries ([Gintis 2008](#_ENREF_13); [Herrmann et al. 2008](#_ENREF_17)). In our study, by focussing on a society culturally very different from any one found in the western and other parts of the world, we expected to obtain some novelty. In this case, the cooperation and defection results showed a trend so markedly different between the genders that went beyond expectations.

The country of China itself exempted us to ponder about confounding elements affecting individuals with different religious beliefs and with less conformist attitudes ([Jenkins 2002](#_ENREF_19)), not in line with an established Confucian mind-set ([Henrich 2014](#_ENREF_14); [as also recently noted by Talhelm et al. 2014](#_ENREF_27)). We in fact did not find much inter-individual variation across our population sample, a prominent element of more individualistic societies ([Camerer & Fehr 2006](#_ENREF_3); [Gächter & Thoeni 2011](#_ENREF_12)) that can cause variation leading to the instability of the overall cooperation ([McNamara & Leimar 2010](#_ENREF_23)).

Scientists, who study evolution of cooperation in computer labs by the means of behavioural economics, are to a certain extent aware of the limitations of such studies ([Gächter et al. 2010](#_ENREF_11); [Levitt & List 2007](#_ENREF_21)). By using computer strategic games to study human decision-making, we tested a model using a relatively easy-to-implement experiment. This setting offers many advantages, namely that the subjects spontaneously chose their preferred strategy instead of stating intentions as in questionnaires. In our study, we exclusively focused on university students as representative subjects and asked them to prompt us with responses originating from their economic decision making rational motive. Comparing their behaviour to individuals of broader ages and tested in the field could be debatable ([Henrich et al. 2006](#_ENREF_16)). Notwithstanding, behavioural economics in the laboratory still provide us with the most effective tool and larger datasets that we can compare our results with ([Balliet et al. 2011](#_ENREF_1)).

Although it was known that women tend to be more risk averse than men in their economic decision making process ([Croson & Gneezy 2009](#_ENREF_9)), only very recently Chinese men and women were tested for their propensity to gamble money. Specifically, Lam ([2014](#_ENREF_20)) found that Chinese women are more risk averse than Chinese men. The effect was nevertheless not dissimilar to the general trend found in other women around the globe ([Charness & Gneezy 2012](#_ENREF_5)). This economic decision element does not therefore justify our peculiar result compared to all the other societies analysed for their cooperation tendency ([Balliet et al. 2011](#_ENREF_1)).

Next, we give more detailed evidence of societal consequences raised by the gender gap found in China. In 1978, Chinese women workers accounted for 33% of the total number of employees; in 2000, the figure raised to 38% ([Chinese National Bureau of Statistics, Zhang 2004](#_ENREF_35)). In 2011 there was a steep growth reaching 64% ([International Labour Organization 2012](#_ENREF_18)). Despite the increase in women occupancy the wage gap widened significantly, decreasing from 77.5% to 70% of the men salary in the same time period ([Zhang 2004](#_ENREF_35)). Based on a survey conducted on urban residents, it was found that the average woman salary was only 84%, 82% and 76% of the man average salary in the years 1987, 1996, and 2004 ([data of the State Statistics Bureau of China, Wei 2008](#_ENREF_31)). We bring forward another figure, more closely relevant to the students’ life. In 2007, six months after graduation, the wage discrepancy between the genders was over 17%, of which it was calculated mostly attributable to gender discrimination again ([China economics annual conference 2009](#_ENREF_6)).

Asymmetries play an influential role and drive the evolution of cooperation in several model species and not just humans ([Cox et al. 2013](#_ENREF_7); [Wang et al. 2010](#_ENREF_29); [Wang & Shi 2010](#_ENREF_30)). With this study we shed light on the importance that such phenomenon plays in the culturally peculiar, traditional Chinese society. Especially so in this inclusive and holistic society, the role that culture seems to express is at first contradictory. The divisionary effect resulting from the higher cooperation of men versus the higher defection of women is not immediate to understand. In fact, in this context, a discriminative culture in respect to gender roles seems to have shaped the archetypal and evolutionary conserved decision making strategies into what we see today here ([as also noted in other cultural contexts by Gächter et al. 2010](#_ENREF_11)).

We do not know whether with time, the steep economic growth of this country will modify this gender difference effect. It will be interesting to see whether a millenary tradition of Confucian philosophy can be modified by economic societal changes.

## References

Balliet, D., Li, N. P., Macfarlan, S. J. & Van Vugt, M. 2011. Sex differences in cooperation: a meta-analytic review of social dilemmas. *Psychological bulletin*, 137, 881

Bian, Y. 2002. Chinese social stratification and social mobility. *Annual review of sociology*, 91-116

Camerer, C. F. & Fehr, E. 2006. When Does "Economic Man" Dominate Social Behavior? *Science*, 311, 47-52

Cárdenas, J.-C., Dreber, A., von Essen, E. & Ranehill, E. 2014. Gender and Cooperation in Children: Experiments in Colombia and Sweden. *PLoS ONE*, 9, e90923, 10.1371/journal.pone.0090923.

Charness, G. & Gneezy, U. 2012. Strong evidence for gender differences in risk taking. *Journal of Economic Behavior & Organization*, 83, 50-58

China economics annual conference, c. f. p. 2009. In Chinese [Gender Wage Differentials and Discrimination in the Labor Market for Chinese College Graduates].

Cox, J. C., Ostrom, E., Sadiraj, V. & Walker, J. M. 2013. Provision versus appropriation in symmetric and asymmetric social dilemmas. *Southern Economic Journal*, 79, 496-512

Cox, T. H., Lobel, S. A. & McLeod, P. L. 1991. Effects of ethnic group cultural differences on cooperative and competitive behavior on a group task. *Academy of management journal*, 34, 827-847

Croson, R. & Gneezy, U. 2009. Gender differences in preferences. *Journal of Economic Literature*, 47, 1-27

Davies, J., Lluberas, R. & Shorrocks, A. 2013. Credit Suisse Global Wealth Databook 2013. CREDIT SUISSE Research Institute.

Gächter, S., Herrmann, B. & Thöni, C. 2010. Culture and cooperation. *Philosophical Transactions of the Royal Society B: Biological Sciences*, 365, 2651-2661

Gächter, S. & Thoeni, C. 2011. Micromotives, microstructure, and macrobehavior: the case of voluntary cooperation. *The Journal of Mathematical Sociology*, 35, 26-65

Gintis, H. 2008. Punishment and Cooperation. *Science*, 319, 1345-1346, 10.1126/science.1155333.

Henrich, J. 2014. Rice, Psychology, and Innovation. *Science*, 344, 593-594

Henrich, J., Heine, S. J. & Norenzayan, A. 2010. Most people are not WEIRD. *Nature*, 466, 29-29

Henrich, J., McElreath, R., Barr, A., Ensminger, J., Barrett, C., Bolyanatz, A., Cardenas, J. C., Gurven, M., Gwako, E., Henrich, N., Lesorogol, C., Marlowe, F., Tracer, D. & Ziker, J. 2006. Costly punishment across human societies. *Science*, 312, 1767-1770

Herrmann, B., Thöni, C. & Gächter, S. 2008. Antisocial Punishment Across Societies. *Science*, 319, 1362-1367, 10.1126/science.1153808.

International Labour Organization. 2012. Global Employment Trends for Women 2012. International Labour Organization.

Jenkins, T. N. 2002. Chinese traditional thought and practice: lessons for an ecological economics worldview. *Ecological Economics*, 40, 39-52, <http://dx.doi.org/10.1016/S0921-8009(01)00263-4>.

Lam, D. 2014. Gender Differences in Risk Aversion Among Chinese University Students. *Journal of Gambling Studies*, 1-11, 10.1007/s10899-014-9492-z.

Levitt, S. D. & List, J. A. 2007. What do laboratory experiments measuring social preferences reveal about the real world? *The journal of economic perspectives*, 153-174

Markus, H. R. & Kitayama, S. 1991. Culture and the self: Implications for cognition, emotion, and motivation. *Psychological review*, 98, 224

McNamara, J. M. & Leimar, O. 2010. Variation and the response to variation as a basis for successful cooperation. *Philosophical Transactions of the Royal Society B: Biological Sciences*, 365, 2627-2633, 10.1098/rstb.2010.0159.

Nee, V. 1996. The emergence of a market society: Changing mechanisms of stratification in China. *American Journal of Sociology*, 908-949

Nisbett, R. E., Peng, K., Choi, I. & Norenzayan, A. 2001. Culture and systems of thought: holistic versus analytic cognition. *Psychological review*, 108, 291

Oyserman, D., Coon, H. M. & Kemmelmeier, M. 2002. Rethinking individualism and collectivism: evaluation of theoretical assumptions and meta-analyses. *Psychological bulletin*, 128, 3

Talhelm, T., Zhang, X., Oishi, S., Shimin, C., Duan, D., Lan, X. & Kitayama, S. 2014. Large-Scale Psychological Differences Within China Explained by Rice Versus Wheat Agriculture. *Science*, 344, 603-608

Walder, A. G. 2002. Markets and income inequality in rural China: Political advantage in an expanding economy. *American Sociological Review*, 231-253

Wang, R., He, J., Wang, Y., Shi, L. & Li, Y. 2010. Asymmetric interaction will facilitate the evolution of cooperation. *Science China Life Sciences*, 53, 1041-1046, 10.1007/s11427-010-4016-2.

Wang, R. & Shi, L. 2010. The evolution of cooperation in asymmetric systems. *Science China Life Sciences*, 53, 139-149

Wei, C. 2008. In Chinese [Study of the gender earnings gap between Chinese cities]. *Statistical research*, 25, 54-58

Yang, K.-S. 1995. Chinese social orientation: An integrative analysis. *Chinese societies and mental health*, 19-39

Yang, K.-S. 1996. The psychological transformation of the Chinese people as a result of societal modernization.

Yifei, S. 2011. China in the "Post-Patriarchal Era". *Chinese Sociology & Anthropology*, 43, 5-23

Zhang, D. 2004. In Chinese [Study of the gender wage gap in the market]. *China Population Science*, 1, 32-42
